# Current scientific evidence for why periodontitis should be included in diabetes management

**DOI:** 10.3389/fcdhc.2023.1257087

**Published:** 2024-01-11

**Authors:** Wenche Sylling Borgnakke

**Affiliations:** ^1^ Department of Periodontics and Oral Medicine, School of Dentistry, University of Michigan, Ann Arbor, MI, United States; ^2^ Department of Periodontics and Preventive Dentistry, School of Dental Medicine, University of Pittsburgh, Pittsburgh, PA, United States

**Keywords:** early diagnosis, glycated hemoglobin, health care costs, interprofessional relations, periodontal diseases, prevention and control, referral and consultation, surgical clearance

## Abstract

This Perspective provides a brief summary of the scientific evidence for the two-way links between periodontal diseases and hyperglycemia (diabetes mellitus [DM] and pre-DM). It delivers in a nutshell current scientific evidence for manifestations of hyperglycemia on periodontal health status and effects of periodontal diseases on blood glucose levels and in turn incidence, progression, and complications of diabetes. Of outmost importance is presentation of scientific evidence for the potential of routine periodontal treatment to lower blood glucose levels, providing a novel, economical tool in DM management. Non-surgical periodontal treatment (“deep cleaning”) can be provided by dental hygienists or dentists in general dental offices, although severe cases should be referred to specialists. Such therapy can decrease the costs of DM care and other health care costs for people with DM. The great importance of a healthy oral cavity free of infection and subsequent inflammation – especially periodontitis that if untreated will cause loosening and eventually loss of affected teeth – has largely gone unnoticed by the medical community as the health care curricula are largely void of content regarding the bi-directional links between oral health and systemic health, despite elevation of blood glucose levels being an integral part of the general systemic inflammation response. The importance of keeping disease-free, natural teeth for proper biting and chewing, smiling, self-esteem, and pain avoidance cannot be overestimated. Medical and dental professionals are strongly encouraged to collaborate in patient-centered care for their mutual patients with – or at risk for – hyperglycemia.

## 1 Introduction

Periodontitis and diabetes mellitus (DM) often co-occur: People with periodontitis have greater risk of hyperglycemia and those with hyperglycemia have greater risk of periodontitis. This is expected because these conditions are both chronic, inflammation-associated diseases that share the same modifiable (1) and non-modifiable risk factors (2) as other non-communicable inflammation related chronic diseases (3, 4) and both are independently associated with DM complications and mortality (5, 6). Moreover, research has demonstrated causal effects between hyperglycemia and periodontitis in a dose-response relationship in both directions (two-way or bi-directional relationship) (7–14). DM and periodontitis are prevalent globally and consume huge human, medical/dental, and financial resources, described in the **Supplementary Material**.

## 2 Periodontal diseases

Periodontal diseases affect the soft and hard tissues surrounding the teeth (15).

### 2.1 Gingivitis

Gingivitis is mostly dental plaque (bacteria and food remnants attached to teeth)-induced inflammation of the soft gingiva (gum tissue surrounding the teeth) and is reversible by home oral hygiene measures (tooth brushing, flossing, etc.) (16–18). Dental plaque can calcify above and below the gum line, causing inflammatory responses in the surrounding tissues due to both its hardness and to harboring microbes lodged in niches in its rough surface, causing inflammation (17–19).

#### 2.1.1 Effect of hyperglycemia: biology

Nonetheless, in hyperglycemia, gingivitis can occur without dental plaque initiation (8, 9, 20–23) because excess blood glucose is toxic and induces mitochondrial stress and respiratory bursts in inflammatory stress that in turn activates proinflammatory mediator cascades (24). Additionally, advanced glycation end‐products (AGEs) and their receptors (RAGE) on the cell surface induce proinflammatory signaling cascades (10, 25).

#### 2.1.2 Effect of hyperglycemia: prevalence

Persons with DM have much greater prevalence of gingivitis than non-DM (26).

### 2.2 Chronic periodontitis

#### 2.2.1 Biology

In especially susceptible individuals with compromised immune systems, gingivitis can progress to periodontitis, a chronic, irreversible breakdown of both gingiva and jaw bone, the intensity and severity of which depend more on the host responses than on specific bacterial agents (27, 28). Deepened spaces (pockets) around the teeth harbor at least 700 (~1,000)? difference species of bacteria plus virus, fungi, and archaea, several of which easily penetrate the inflamed, swollen gingival tissues and travel everywhere in the host via all body fluids (blood, saliva, lymph, tears, urine) and along nerves (29). *Porphyromonas gingivalis (Pg)* (30), “*a master of immune subversion”* (31) has developed sophisticated mechanisms to avoid detection/dissolution by the host’s immune system and survives during its body-wide travels (29, 32), resulting in *Pg* and its byproducts (polysaccharides) causing local and systemic inflammation (27–29).

#### 2.2.2 Effect of hyperglycemia: biology

The structure and relative abundance (composition) of bacteria in the subgingival microbiome are significantly different in normoglycemia and hyperglycemia (33–40) and predicts glucose change in non-DM (41).

Hyperglycemia leads to inflammatory response disturbances (42), enhancing pro-inflammatory cytokines and matrix metalloproteinases (MMP) expression (35), so the periodontal tissue metabolism adversely affects blood vessels and promotes periodontitis, which develops more rapidly and more intensely (43), with hyperglycemia enhancing its progression (44, 45), even at pre-DM levels (46). Importantly, hyperglycemia severity, not the DM diagnosis, affects the periodontium (47, 48).

#### 2.2.3 Effect of hyperglycemia: prevalence

Periodontitis was proclaimed DM’s 6^th^ complication in 1993 (49)–without much attention from the medical community. Hyperglycemia is a risk driver for incident periodontitis (1, 5–8, 22, 23, 45, 50–52), and increases its severity (37) (**Supplementary Material**).

### 2.3 Tooth loss due to periodontitis

If left untreated, periodontitis may result in loosening and eventual loss of the tooth (15), being one of 2 major causes (with caries) of tooth loss in adults (53–57).

#### 2.3.1 Effects of hyperglycemia and tooth loss

People with DM have an impaired immune system and experience 1.3-5-fold greater tooth loss (53, 58–63), including losing all teeth (edentulism) (61, 64, 65), compared to those with normoglycemia. Loose or missing teeth decrease quality of life (66–72) and impair masticatory function (73, 74), preventing biting/chewing crisp or hard healthy diet components recommended (74, 75), leading to consumption of soft food items typically containing few fibers and nutrients (76, 77), laden with sugar, fat, and salt.

#### 2.3.2 Tooth substitutes: dental implants and removable prostheses

A crown-restored dental implant placed in the jaw bone may replace a lost tooth, but implants suffer from diseases parallel to the natural teeth, namely the reversible peri-implant mucositis and the irreversible peri-implantitis, the latter being extremely challenging to treat successfully. A more economical alternative is a removable prosthesis that rests on some natural teeth. Both options are costly and beyond reach for many DM patients.

#### 2.3.3 Effect of hyperglycemia: peri-implantitis

The composition of the microbiome around implants is significantly different from that around teeth, especially in the deep peri-implant pockets (78). Those with hyperglycemia experience much greater risk for peri-implantitis (79–83)–including metabolic syndrome with 15-fold risk reported (84)–and for implant loss (79), with failure rates greater in hyperglycemia than in other diseases (85, 86), and greater in D1T than in D2T (79).

## 3 DM/hyperglycemia

DM is a carbohydrate-lipid metabolic disorder caused by insufficient insulin production, insensitivity to normal amounts of insulin, or both, resulting in abnormally high blood glucose levels (hyperglycemia, dysglycemia) (85) (**Supplementary Material**).

### 3.1 Effect of periodontitis: biology

The chronic, repetitious discharge of periodontal microbes and their byproducts into the bloodstream causes inflammatory markers to circulate and hence create or exacerbate insulin resistance (87–91) and DM complications (92, 93).

### 3.2 Effects of periodontitis: incidence of T2D, glycemic control in existing DM, and DM complications

Periodontitis negatively influences glucose control in existing T2D, contributes to incident T2D and to DM complications in a dose-response manner (87–89, 94).

## 4 Mechanisms underlying the bidirectional links between periodontitis and DM

**Figure 1:**
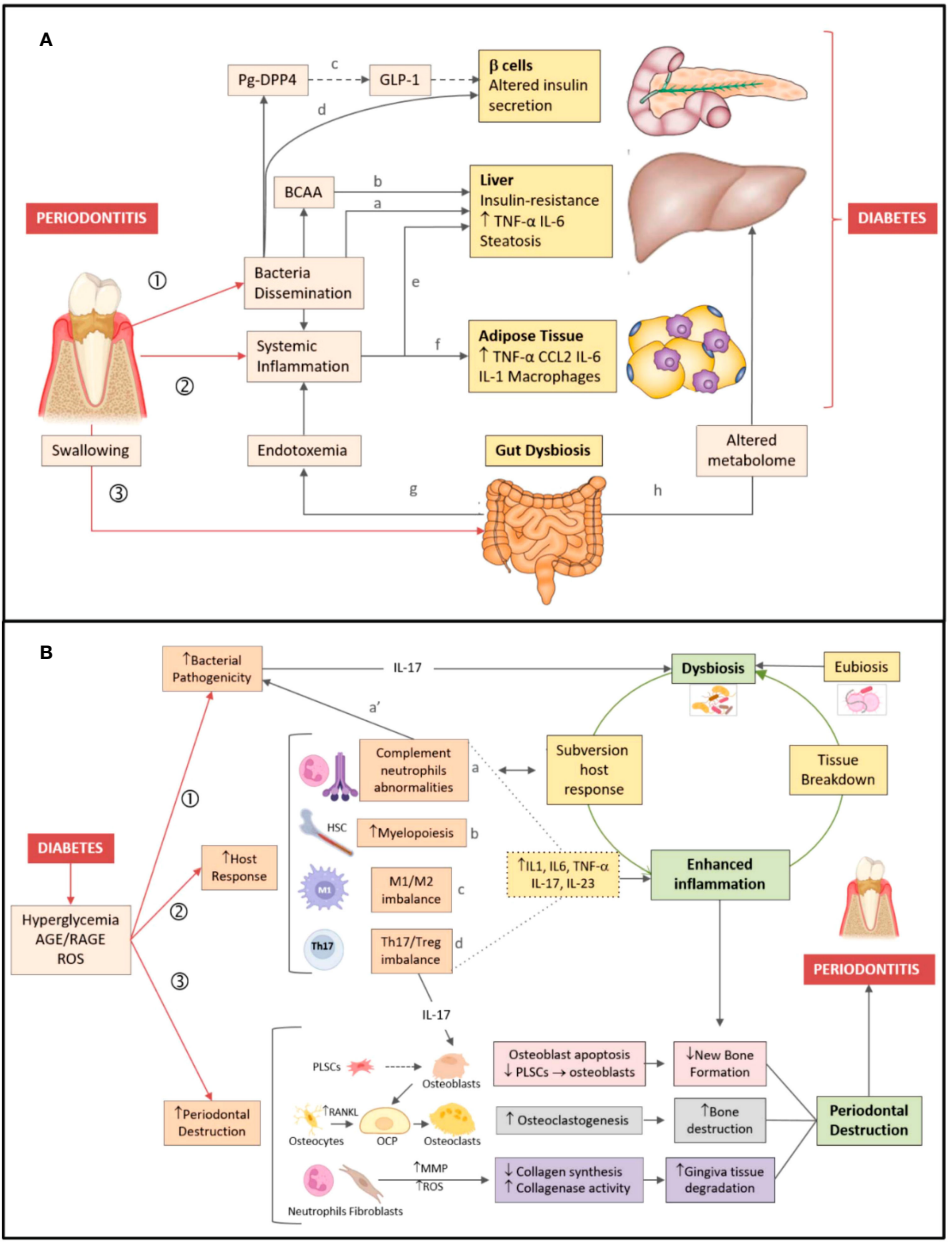
Bidirectional relationship between periodontitis and diabetes. **(A)** Periodontitis → diabetes direction. Periodontitis favors development/worsening of type 2 diabetes by three major mechanisms: (1) Dissemination of periodontal bacteria/bacterial products into the bloodstream. Bacteria/bacterial products can induce insulin resistance (a) by inhibiting hepatic glycogen synthesis, increasing hepatic gluconeogenesis, and (b) blocking the insulin receptor substrate via production of branched-chain amino acids (BCAA). (c) Dipeptidyl peptidase-4 (DPP4) produced by *P. gingivalis* (Pg-DPP4) can reduce glucose-induced insulin production by enhancing glucagon-like peptide 1 (GLP-1) degradation. (d) *P. gingivalis* may alter insulin production by inducing β cell dedifferentiation. (2) Induction/magnification of systemic inflammation, favoring both (e) hepatic and (f) adipose tissue insulin resistance. (3) Gut dysbiosis induced by swallowed periodontal bacteria, favoring both (g) endotoxemia and (h) changes in the blood metabolome. **(B)** Diabetes → periodontitis direction. Pathogenesis of periodontitis is depicted on the right-hand side of the figure. Dysbiosis, inflammation, and destruction of the periodontium *(green boxes)* are characteristic features of periodontitis. Dysbiotic bacteria reduce the efficacy of the host immune response, while fueling inflammation *(open green arrow)*. In turn, inflammation-induced tissue breakdown favors dysbiosis *(closed green arrow)* closing the vicious cycle. Mechanisms linking diabetes to periodontitis are shown on the left-hand side of the figure. Diabetes favors development/worsening of periodontitis by three major mechanisms: (1) Increasing periodontal dysbiosis and bacterial pathogenicity via IL-17; (2) Enhancing the host response to the bacterial challenge. Diabetes (a) alters complement and neutrophil function (which also affects susceptibility to infection a’), (b) increases myelopoiesis, enhances (c) the M1/M2 macrophage ratio, (d) the Th17/Treg lymphocyte ratio, thus raising inflammatory cytokines levels (dotted lines) and fueling inflammation. (3) Increasing periodontal destruction. Diabetes reduces new bone formation by enhancing apoptosis of bone-forming cells and by lowering periodontal ligament stem cells (PLSCs) proliferation and differentiation in osteoblasts *(pink boxes)*. Diabetes enhances osteoclastogenesis by increasing RANKL release by osteocytes/osteoblasts, leading to osteoclast precursor (OCP) differentiation in osteoclasts *(grey boxes)*. Diabetes augments gingiva tissue degradation by increasing release of metalloproteinases (MMP) and reactive oxygen species (ROS) by neutrophils and fibroblasts *(violet boxes)* (11). Figure and legend created and copyrighted by Barutta et al. in 2022 (11), and reproduced here without any changes. https://www.ncbi.nlm.nih.gov/pmc/articles/PMC8774037/figure/biomedicines-10-00178-f001/. This article is an open access article distributed under the terms and conditions of the Creative Commons Attribution 4.0 International (CC BY) license (https://creativecommons.org/licenses/by/4.0/).

**Figure 1** shows conceptual models of the mechanisms underlying the two-way effects linking periodontitis and DM (11), shown differently in References (7, 12, 13).

## 5 Effects of periodontal treatment

### 5.1 Blood glucose level

Removal of soft and hardened plaque (calculus, “tartar”) above and below the gum line (non-surgical periodontal treatment, scaling and root planing, a. k. a. “deep cleaning”) can decrease HbA1c levels with at least 26 prior systematic reviews with meta-analyses and umbrella reviews calculating and reporting such HbA1c decrease to be around 0.5 percentage point from 3 to 12 months after periodontal therapy (7, 13, 95–118). This decrease is in the order of magnitude of the expected effect of adding a second oral anti-diabetic medication to metformin (119) and is hence clinically significant (13, 120). For a clinical perspective, 1 percentage point HbA1c reduction may reduce DM mortality by 21%, myocardial infarction by 14%, and DM microvascular complications by 37% (121). Non-surgical periodontal treatment reduces insulin resistance, improving insulin sensitivity in T2D (122).

Greater effect is seen with higher baseline HbA1c level (98), but decreases with increasing age (123), due to inflammaging or immunosenescence caused by diminishing immune defense efficiency in older ages (124–133).

### 5.2 Inflammatory markers

Periodontal treatment decreases levels of several inflammatory biomarkers, many of which in turn are risk factors for DM complications like atherosclerosis and myocardial infarction, such as c-reactive protein (134) interleukin-(IL)-1beta (91), IL-6 (91), tumor necrosis factor-alpha (TNF-alpha) (91). Statistically significant decreases in the concentration of the active inflammatory marker, c-reactive protein, from baseline to each 3-monthly visit up to 1 year upon extraction of terminally periodontally diseased teeth and scaling and root planing the remaining dentition are reported (134).

## 6 Interprofessional collaboration

The call for patient centered, interprofessional, transdisciplinary, and interdisciplinary collaboration is increasingly loud in an abundance of scientific papers and guidelines (4, 13, 135–147). Please see the APPENDIX and online-only **Supplementary Material**.

### 6.1 Hyperglycemia/DM in the dental setting

Because almost half of people with DM (148) and 90% of those with pre-DM are unaware thereof (148), the dental setting can be helpful in identifying these individuals, which is urgent for early diagnosis and prevention of DM complications (148, 149) Globally, dental professionals are increasingly aware of the two-directional association between hyperglycemia and periodontitis and they include DM in anamneses (150). They support chair-side screening for hyperglycemia despite citing barriers like time constraint, patient cooperation, cost/insurance coverage, and lack of equipment (150).

Two systematic reviews and meta-analyses of studies involving finger prick blood sampling among dental patients denying hyperglycemia calculated a T2D prevalence of 1.7%-46.4% and of pre-DM 23.3%-68.0% (151, 152). Gingival crevicular blood from the pocket is also a valid screening tool (153–157) because of its high correlation glucose concentration with serum. Hyperglycemia screening in the dental setting is found cost-effective (158, 159), and general physicians are receptive to receive referrals from dentists (160). Few teeth/edentulism and periodontitis are significantly associated with undiagnosed DM (63, 161) and hence predict potential hyperglycemia.

DM is such a strong risk driver for periodontitis that DM is incorporated in the grading of the stages of the most recent *European Federation of Periodontology (EFP)/American Academy of Periodontology (AAP)* periodontitis classification (162, 163). The Finnish Diabetes Risk Score (FINDRISC) and a periodontal disease risk score are significantly linearly correlated (164).

*Federation Dentaire International (FDI)* dedicated its entire 2017 World Forum to periodontitis and created action items to enhance global awareness of its connection to DM and other non-communicable diseases to promote general health (165) in line with the *United Nations* and *World Health Organization* (166).

A crude search on “diabetes” at the *American Dental Association (ADA)’s* home page (https://www.ada.org/) resulted in 93 hits.

The *American Academy of Periodontology (AAP)* recently created a 38-page report and 3 infographics that includes DM (167).

### 6.2 Periodontitis in DM management in the medical setting

The *International Diabetes Federation (IDF)* acknowledged the importance of good oral health in DM management by including an oral health section in the 9^th^ edition of the *IDF Diabetes Atlas* (168), along with more detailed description in its scientific journal (120). Importantly, *IDF* and *EFP* published a consensus document with guidelines upon a joint workshop published in the respective organizations’ journals (5, 6).

In its 3rd edition of the mammoth work, “*Diabetes in America*” (169), the *National Institute of Diabetes and Digestive and Kidney Diseases (NIDDK)* included with great enthusiasm a 51-page long (*versus* 6 pages in the 2^nd^ edition from 1995 (170)) chapter titled *31. Oral health and diabetes*” (7), in which the periodontitis-DM links are prominently described, both by existing literature and by new analyses of National Health and Nutrition Examination Survey (NHANES) data specifically for this chapter.

A search for “oral health” at the *American Medical Association (AMA)’s* homepage (https://www.ama-assn.org/) turned up 2 hits, one of which links to a paper by physician Hugh Silk, a long-time, avid advocate for integration of primary care and oral health (139, 171–176).

#### 6.2.1 American Diabetes Association (AD[M]A)

In contrast to all the global medical and dental organizations’ websites, there is no mention of oral health, let alone periodontitis, anywhere at AD[M]A’s website–despite:

1) periodontitis is an established risk factor for DM;
2) periodontitis is a DM complication;
3) international acceptance of the strong, current scientific evidence for the bi-directional causal relationship between periodontitis and hyperglycemia in both Medicine and Dentistry (5–7, 13, 120);
4) extraction of severely periodontally diseased teeth can decrease HbA1c concentration clinically significantly;
5) non-surgical periodontal therapy can decrease HbA1c concentration clinically significantly;
6) periodontal therapy decreases costs for both DM and general care, hospitalizations, and pharmaceuticals in patients with DM;
7) AD[M]A emphasizes a holistic approach to DM care, including in its consensus statement with the *European Association for the Study of Diabetes (EASD)* (177); and
8) initially poorly controlled DM patients improve their glucose control with dental visits (178).

##### 6.2.1.1 “Standards of care in diabetes”

Every year, *AD(M)A* updates its “*Standards of Care in Diabetes*” guidelines for diagnosing and treating DM, published as a supplement to the January issue of its journal Diabetes Care. The 2023 edition consists of 20 individual articles totaling 302 pages (179). Regrettably, it still cites dated oral health related references published from 2011 to 2019 (180–183) under *“2. Classification and Diagnosis of DM”* (85) (exactly the same 4 references as in 2022 (184)) and in 2006-2018 (14, 88, 113, 185–187) under *“4. Comorbidities”* (188), respectively. The 2023 Writing Group even had the audacity to totally deny any relationship between DM and periodontitis or any other oral disease, as they write: *“ASSESSMENT OF COMORBIDITIES: Besides assessing diabetes-related complications, clinicians and people with diabetes need to be aware of common comorbidities that affect people with diabetes and that may complicate management* (14, 88, 113, 185–187)*. Diabetes comorbidities are conditions that affect people with diabetes more often than age-matched people without diabetes”* (188). Note: There is not even any mention of oral diseases, which the attentive reader needs to discover by consulting the bibliography. In “*Table 4.4—Referrals for* ***initial*** *care management,”* it still says “*Dentist for comprehensive dental and periodontal examination”* (188), identically to in 2022 (189). The relevant text passage reads: “*People with diabetes should receive recommended preventive care services (e.g., immunizations, cancer screening); smoking cessation counseling; and ophthalmological, dental, and podiatric referrals, as needed*” (188).

Clearly, periodontitis is not considered part of ongoing DM management (179).

##### 6.2.1.2 Why?

A crude PubMed search: “diabetes and (periodontitis or ‘oral health’)” resulted in 22,149 hits. Why does *AD[M]A* totally and purposefully deny the large body of scientific literature regarding roles of periodontitis and any other inflammatory oral disease, whose treatment provides a scientifically sound and uncomplicated, straightforward tool to prevent and manage hyperglycemia DM?

Due to the global reach of *AD[M]A*, this is extremely detrimental to both the global medical community and the patients they serve worldwide, especially in under-resourced settings.

###### 6.2.1.2.1 Incorporation of periodontitis into medical management of DM

Despite primary care providers expressing positive attitudes towards medical-dental care models, integrated care is rarely practiced (190–192), with Marshfield Clinic Health System (MCHS) being an exception (146, 191, 193, 194).

###### 6.2.1.2.2 Lack of incorporation of periodontitis into medical management of DM

The degree of tooth loss decreases with increasing numbers of dental visits (61). Despite international medical/endocrinologic guidelines (except the AD[M]A’s) recommending at least annual dental checkups, DM patients are globally and consistently reported to have fewer dental checkup visits than in normoglycemia (59, 195–202).

##### 6.2.1.3 Periodontal care reduces cost of medical care in DM

Persons with DM have greater dental (203) and medical care costs (204, 205) than their normoglycemic peers. (**Supplementary Material**). However, periodontal therapy and maintenance visits decrease expenses for both DM care (206–209) and other medical care, including pharmaceuticals and hospitalizations (206, 207, 210–213).

##### 6.2.1.4 Malpractice lawsuits

Due to the impaired immune response in DM, “dental clearance” prior to any invasive procedure may be prudent to minimize the risk for periodontitis/loose teeth serving as reservoirs for bacteria causing infection in remote locations (29, 214).

Patients are increasingly aware of the importance of periodontitis for general health and of the potentially fatal consequences of its neglect. For example, when the DM complication myocardial infarct occurs, medical care providers may experience legal consequences by the survivor or the deceased’s estate for neglecting referral for proper dental examination and therapy – just like the dentists neglecting examining for and managing periodontitis (215). Hence, legal liability is another reason for medical and dental care professionals to take periodontitis seriously.

## 7 Discussion

In a nutshell: Periodontitis and diabetes are strongly linked in a two-way causal relationship. Hyperglycemia negatively affects the periodontal soft and hard tissues, facilitating their breakdown and impairing their healing. Chronic periodontitis-associated bacteremia and subsequent local and systemic inflammation increase blood glucose levels, which in turn can be decreased by periodontal treatment by a clinically relevant magnitude around 0.5 percentage point HbA1c after up to 12 months of maintenance visits after the initial therapy.

Therefore, it would be prudent for medical and dental professionals to collaborate in patient-centered, interprofessional management teams caring for their mutual patients with hyperglycemia.

## Data availability statement

The original contributions presented in the study are included in the article/**Supplementary Material**. Further inquiries can be directed to the corresponding author.

## Author contributions

WSB: Conceptualization, Funding acquisition, Methodology, Writing – original draft, Writing – review & editing.
