# Supplementary material for: Current scientific evidence for why periodontitis should be included in diabetes management
Source: Front Clin Diabetes Healthc. 2024 Jan 11;4:1257087. doi: 10.3389/fcdhc.2023.1257087 (PMC10809181; doi:10.3389/fcdhc.2023.1257087)
Supplement: Supplementary file 1 [file DataSheet_1.pdf]

APPENDIX

GUIDELINES FOR MEDICAL & DENTAL PROFESSIONALS & THEIR PATIENTS*

5.1 | Guidelines for physicians and other medical health professions for use in diabetes practice

Because of the increased risk for developing periodontitis in patients with diabetes and the negative impact of periodontitis on diabetes control and complications, the following recommendations are made:

- Oral health education should be provided to all patients with diabetes as part of their overall educational programme.
- Patients with all forms of diabetes mellitus should be told that periodontal disease risk is increased, and if untreated, the periodontitis has a negative impact on metabolic control and may also increase the risk of complications of their diabetes such as cardiovascular and kidney disease.
- Patients should be advised that successful periodontal therapy may have a positive impact upon their metabolic control and diabetes complications.
- For people with diabetes, physicians should ask about a prior diagnosis of periodontal disease. If a positive diagnosis has been made, the physician should seek to ascertain that periodontal care and maintenance are being provided.
- Investigating the presence of periodontal disease should be an integral part of a diabetes care visit. People with diabetes should be asked about any signs and symptoms of periodontitis, including bleeding gums during brushing or eating, loose teeth, spacing or spreading of the teeth, oral malodor and/or abscesses in the gums or gingival suppuration.
  - If a positive history is elicited, then a prompt periodontal evaluation should be recommended before their scheduled annual check-up.
  - In the case of a negative history, people with diabetes should be advised to check for the above symptoms, and if a positive sign appears, they should visit their dentist.
- For all people with newly diagnosed diabetes mellitus, referral for a periodontal examination should occur as part of their ongoing management of diabetes. Even if no periodontitis is diagnosed initially, annual periodontal review is recommended.
- For children and adolescents diagnosed with diabetes, annual oral screening is recommended through referral to a dental professional.
- Patients with diabetes who have extensive tooth loss should be encouraged to pursue dental rehabilitation to restore adequate mastication for proper nutrition.
- Patients with diabetes should be advised that other oral conditions such as dry mouth and burning mouth may occur, and if so, they should seek advice from their dental practitioner. Also, patients with diabetes are at increased risk of oral fungal infections and experience poorer wound healing than those who do not have diabetes.
- The physician should liaise with the dentist over diabetes management prior to the oral intervention and/or surgery to avoid hypoglycaemia and to consider its potential impact on the patient's ability to eat (1,2).

5.2 | Guidelines for patients with diabetes at the physician's practice/office

5.2.1 | Why should I have my gums checked?

If your physician has told you that you have diabetes, you should make an appointment with a dentist to have your mouth and gums checked. This is because people with diabetes have a higher chance of getting gum disease. Gum disease can lead to tooth loss and may make your diabetes harder to control. The earlier you seek help, the better the outcome will be.

5.2.2 | What should I look for that may tell me I have problems with my gums?

You may have gum disease if you have ever noticed:

- Red or swollen gums;
- Bleeding from your gums or blood in the sink after you brush your teeth;
- Foul taste;
- Longer looking teeth;
- Loose teeth;
- Increasing spaces between your teeth;
- Calculus (tartar) on your teeth.

If you have noticed any of these problems, it is important to see a dentist as soon as possible.

5.2.3 | Can I have gum disease without these signs being present?

Gum disease may also be present and get worse with no apparent signs to you, especially if you smoke, so even if you do not think you have gum disease now, you should still have annual dental check- ups as part of managing your diabetes. Your dentist will be able to pick up early signs of gum disease.

5.2.4 | What can I do to prevent gum disease?

You need to clean your teeth and gums twice daily at home for a mini-mum of 2 min. Also, cleaning between your teeth daily is important and your oral health professional will show you how to do this. You should visit a dentist as soon as possible for a diagnosis and advice on what you need to do. It is important to keep your mouth as healthy as possible with regular dental care, according to the recommendations of your oral health professional.

5.2.5 | What other problems with my mouth should I be looking for?

If you have diabetes, you may also suffer from dry mouth, burning mouth or poor healing of mouth wounds.

5.3 | Guidelines for oral health professionals for use in dental practice/office for people with diabetes mellitus

- People with diabetes should be advised that they have an increased risk for gingivitis and periodontitis. They should also be told that if they suffer from periodontitis, their glycaemic control may be more difficult to achieve, and they are at higher risk of other complications such as eye, kidney and cardiovascular diseases.
- Collect a careful history to highlight the type of diabetes, duration of the disease, the presence of any complications, diabetes therapy and concomitant therapies, remembering that most people with diabetes are also being treated with anticoagulant/antiplatelet drugs, antihypertensive drugs or lipid-lowering medications.
- Ask the patient how well controlled their diabetes is and when they last had their blood glucose levels checked. Request that patients bring a copy of their last HbA1C result, or that they report their latest results.
- Oral health education should be provided to all patients with diabetes. This should include individualized advice on relevant risk factors, and a tailored oral hygiene regime, including twice-daily brushing, inter-dental cleaning and in some cases the use of adjunctive chemical plaque control, may be appropriate.
- People presenting with a diagnosis of any form of diabetes mellitus should receive a thorough oral examination, which includes a comprehensive periodontal evaluation, to include full-mouth pocket chart and bleeding scores if indicated by periodontal screening.
- If no periodontitis is diagnosed initially, patients with diabetes should be placed on a preventive care regime and monitored regularly for periodontal changes.
- People with diabetes presenting with any acute oral/periodontal infections require prompt oral/periodontal care. If periodontitis is diagnosed, it should be managed without delay.
- Irrespective of the level of diabetes control, non-surgical periodontal therapy should be provided, as this may help to improve glycaemic control.
- Surgical periodontal and implant therapy is not indicated in patients who do not have acceptable diabetes control. In well-controlled patients, the results of surgical interventions are equivalent to patients without diabetes. However, attention should be paid to:
  - people with poorly controlled diabetes, who have an increased risk of postoperative infections;
  - patients managed with insulin or sulfonylureas, when the physician should be consulted about the timing of the planned procedure and a possible change in dosage of therapy to reduce the risk of intraoperative hypoglycaemia.
- People with diabetes who have extensive tooth loss should be encouraged to pursue dental rehabilitation to restore adequate mastication for proper nutrition.
- People with diabetes should also be evaluated for other potential oral complications, including dry mouth, burning mouth, candida infections and dental caries.
- For children and adolescents diagnosed with diabetes, an annual oral screening for early signs of periodontal involvement and dental caries is recommended starting as early as possible.
- Patients who present in the dental surgery/office without a diagnosis of diabetes, but with risk factors for type 2 diabetes should be informed about their risk for having diabetes and referred to a physician for appropriate diagnostic testing and follow-up care.
  - Patients' risk may be screened for using a validated questionnaire (e.g. in a Caucasian population, FindRisc Questionnaire; https://www.mdcalc.com/findrisc-finnish-diabetes-risk-score
  - For oral health professionals with a special interest in diabetes, they may wish to consider screening based upon the recommendations of the American Diabetes Association: https://pubmed.ncbi.nlm.nih.gov/33298413/
  - If symptomatic (polydipsia, polyuria, polyphagia, unexplained weight loss), refer directly to a physician.

5.4 | Guidelines for patients at the dental surgery/office who have diabetes or are found to be at risk of diabetes

- People with diabetes have a higher chance of getting gum disease.
- You may think that you are doing well managing your gum health, but you may not be doing enough because you have an increased risk of gum problems.
- Like diabetes, gum disease is a chronic condition and requires life-long attention and professional care.
- You also need to clean your teeth and gums very carefully at home. Personalized advice will be provided by your oral health professional. This may include:
  - twice-daily brushing with either manual or electric toothbrush
  - cleaning between your teeth using inter-dental brushes where they fit; where they do not fit, then flossing may be useful
  - the use of specific dentifrices and/or mouth rinses with proven activity against dental plaque, if advised by oral health professionals.
- If left untreated, gum disease can lead to tooth loss and may also make your diabetes harder to control.
- Gum disease may be present and get worse with no apparent symptoms to you, so if your dentist told you that you do not have gum disease now, you should still get regular dental check-ups as part of managing your diabetes. Your dentist will be able to pick up early signs of gum disease.
- You may have gum disease if you have ever noticed:
  - Red or swollen gums;
  - Bleeding from your gums or blood in the sink after you brush your teeth;
  - Foul taste;
  - Longer looking teeth;
  - Loose teeth;
  - Increasing spaces between your teeth;
  - Calculus (tartar) on your teeth.
- People with diabetes may also suffer from dry mouth, burning mouth, yeast infections of the mouth or poor healing of mouth wounds.
- Remember to inform your dentist about the outcome of your visits to your doctor and provide an update of the results of your diabetes control and changes in medications.
- It is important to keep your mouth and your whole body as healthy as possible with regular dental and medical care.

REFERENCES:

1. Albert DA, Ward A, Allweiss P, Graves DT, Knowler WC, Kunzel C, et al. Diabetes and oral disease: implications for health professionals. *Ann N Y Acad Sci.* (2012). doi: 10.1111/j.1749-6632.2011.06460.x

2. Al-Harthi LS, Cullinan MP, Leichter JW, Thomson WM. The impact of periodontitis on oral health-related quality of life: a review of the evidence from observational studies. *Aust Dent J.* (2013) 58:274-7; 384. doi: 10.1111/adj.12076

***SOURCE:**

Reference #5:

Sanz M, Ceriello A, Buysschaert M, Chapple I, Demmer RT, Graziani F, et al. Scientific evidence on the links between periodontal diseases and diabetes: Consensus report and guidelines of the joint workshop on periodontal diseases and diabetes by the International Diabetes Federation and the European Federation of Periodontology. *J Clin Periodontol.* (2018) 45:138-49. doi: 10.1111/jcpe.12808.

Republished with permission from John Wiley and Sons, Publishers (License #5585470726560 dated July 10, 2023).
