# Supplementary material for: Current scientific evidence for why periodontitis should be included in diabetes management
Source: Front Clin Diabetes Healthc. 2024 Jan 11;4:1257087. doi: 10.3389/fcdhc.2023.1257087 (PMC10809181; doi:10.3389/fcdhc.2023.1257087)
Supplement: Supplementary file 2 [file DataSheet_2.pdf]

Current Scientific Evidence for Why Periodontitis Should be Included in Diabetes Management

Wenche Sylling Borgnakke DDS MPH PhD[1,2]

[1]Department of Periodontics and Oral Medicine, School of Dentistry, University of Michigan, Ann Arbor, MI, United States

[2]Department of Periodontics and Preventive Dentistry, School of Dental Medicine, University of Pittsburgh, Pittsburgh, Pennsylvania, USA

SUPPLEMENTARY MATERIAL

A) Prevalence and cost of care for periodontitis and diabetes mellitus (DM) **page 1 - 3**

B) How to screen for periodontitis in the medical setting **page 4**

REFERENCES **page 5 - 8**

A) Prevalence and cost of care for periodontitis and diabetes mellitus (DM)

The following information regarding the prevalence and financial costs of treatment/management of periodontitis and DM is provided to illustrate the importance of both diseases.

PERIODONTITIS

Prevalence

While gingivitis affects up to 90% of adults (1-4) and can be reversed by home oral hygiene measures, such as tooth brushing, flossing, and interdental brushing, periodontitis requires professional intervention. As per the Global Burden of Diseases, Injuries, and Risk Factors study (GBD), severe periodontitis is the 6th most prevalent condition in the world (5), affecting about 796 million or 10%-20% of dentate persons globally in 2010 (6, 7). About 1.1 billion people had severe periodontitis in 2019, an increase of 99.0% from 1990 (8, 9), so severe periodontitis continues to present a serious public health problem, especially in less developed regions, with Africa and the age group 50 – 59 years carry the heaviest burden, but with increasing incidence among younger age groups (10). During the 3 decades from 1990 to 2019, population growth caused two-thirds (67.9%) of the increase in number of cases (8).

Severe periodontitis is the 6th most prevalent condition in the world (5-7), including 7.8% of US dentate people (11, 12).

Among dentate US 30-79-year-olds, 42.2% suffer from periodontitis, with 7.8% having severe periodontitis (11, 12).

Effect of hyperglycemia

People with DM in the US have a 40% greater risk for periodontitis than those without (13),with around 50% greater prevalence (12, 13). Periodontitis among seniors aged >65 years with DM, 83.1 % have periodontitis with 10.8% being severe (14).

Costs

The estimated direct costs of dental diseases amounted to $356.80 billion and indirect costs were estimated at $187.61 billion, totaling worldwide costs due to dental diseases of $544.41 billion in 2015 (15).

Periodontitis treatment costs in the US in 2018 were estimated at $3.49 billion with another $150.57 billion in indirect costs mostly due to periodontitis-related edentulism (16). The latter represents on average 0.73% of the annual gross domestic product (GDP).

The corresponding costs in Europe were Euro2.52 billion and Euro156.12 billion, respectively, with the indirect costs amounting to 0.99% of Europe's annual GDP (16)

Periodontitis is associated with greater medical care costs (17), whereas receiving preventive dental care (dental cleaning and non-surgical periodontal therapy) leads to decreased financial costs for outpatient (including emergency department visits for dental issues) and inpatient medical care in general (18-21).

DIABETES MELLITUS (DM)/HYPERGLYCEMIA

Prevalence

The International Diabetes Federation (IDF) estimated the global prevalence of DM in 20-79 year-olds in 2021 was 10.5% (536.6 million) -- that is more than half a billion people -- and is projected to increase to 643 million by 2030 and to 12.2% (783.2 million) in 2045 (22-24), with older age groups suffering most.

In the US, about 37.3 million people have DM of whom 8.5 million (22.8%) are undiagnosed (25). Additionally, 96 million adults have pre-DM, including 26.4 million (48.8%) of seniors >65 years (25).

Costs

IDF estimated the DM-related global health care costs at USD 966 billion in 2021 and USD 1.054 trillion by 2045 (22, 23).

The estimate global cost of diabetes for 2015 was US 1.31 trillion (1.8% of global GDP, of which indirect costs accounted for 34.7% with North America being the most affected region relative to GDP and also the largest contributor to global absolute costs.(26).

In the US, DM care increased from $37 costs billion in 1996 to $101 billion in 2013 (27). The most recent total estimated cost of diagnosed diabetes in 2017 was $327 billion, including $237 billion in direct medical costs and $90 billion in reduced productivity (DM-related morbidity and premature mortality), representing about 25% of all health care costs, with half of that for DM care (28). Additional costs are

USD 31.7 billion for undiagnosed diabetes, USD 43.4 billion for prediabetes, and nearly USD 1.6 billion for GDM, totaling USD 404 billion (29).

According to the American Diabetes Association (AD(M)A), people with diagnosed DM in the US incur average medical expenditures of approximately USD 16,750 per year, of which approximately USD 9,600 is attributed to diabetes care (28). This is similar to the IDF's estimate of USD 8,208.90 for the North America and Caribbean region (22).

The mean medical expenditures were 2.3 times greater than in those not suffering from DM (28, 30).

The median US state medical care expenditure was USD 18,248 (range: USD 15,418 - USD 30,915) per person with diabetes (31), with direct medical care costs of USD 8,544 (range: USD 6,591 - USD 12,953) and indirect costs USD 9,672 (range: USD 7,133- USD 17,962) (31). Prevention of DM can decrease theses costs considerably (32, 33).

B) How to screen for periodontitis in the medical setting

Because the relevant topics are not generally included in the curricula for medical health care professionals, they are often unaware of the important role periodontitis plays in DM management, or feel unqualified to assess their patients' periodontal health status (34, 35).

Periodontitis may be symptomless until the tooth is loose (36), at which time it is too late to salvage it. However, all health care providers recognize inflammation and suppuration, which signs also are displayed by gingivitis and periodontitis. Here follow verbatim the items for the patient to watch out for listed in the 2018 IDF/European Federation of Periodontology (EFP) consensus document published simultaneously in the two organizations' scientific journals, Diabetes Research and Clinical Practice and Journal of Clinical Periodontology, respectively (37, 38):

- *Red or swollen gums;*
- *Bleeding from your gums or blood in the sink after you brush your teeth;*
- *Foul taste;*
- *Longer looking teeth;*
- *Loose teeth;*
- *Increasing spaces between your teeth;*
- *Calculus (tartar) on your teeth*.

Responses to self-report questionnaire items regarding a patient's periodontal health have good correlation with the clinical status (39-41), except when using non-validated questions (42). The following validated, easily answerable questions for self-report were developed by the Centers for Disease Control and Prevention (CDC)/American Academy of Periodontology (AAP) workgroup (43-45):

- *Do you think you might have gum disease?*
- *Overall, how would you rate the health of your teeth and gums?*
- *Have you ever had treatment for gum disease such as scaling and root planing, sometimes called "deep cleaning"?*
- *Have you ever had any teeth become loose on their own, without an injury?*
- *Have you ever been told by a dental professional that you lost bone around your teeth?*
- *During the past three months, have you noticed a tooth that doesn't look right?*
- *Aside from brushing your teeth with a toothbrush, in the last seven days, how many times did you use dental floss or any other device to clean between your teeth?*
- *Aside from brushing your teeth with a toothbrush, in the last seven days, how many times did you use mouthwash or other dental rinse product that you use to treat dental disease or dental problems?*

These questions were validated in several countries in several languages, including in Japanese adults (39); and based on these items (43-45), a Dutch easy and quick, free online screening tool was developed for the medical settings without an oral examination (46-50). Importantly, general physicians are obligated to screen their patients for periodontitis in The Netherlands (47). A 3-item question (51) and a 7-item tool also screen for periodontitis in non-dental settings (52). Finally, self-reported bleeding on brushing (BoB) is correlated with clinically assessed bleeding on probing (BoP) (53).

REFERENCES

1. Elias-Boneta AR, Toro MJ, Rivas-Tumanyan S, Rajendra-Santosh AB, Brache M, Collins CJ. Prevalence, Severity, and Risk Factors of Gingival Inflammation in Caribbean Adults: A Multi-City, Cross-Sectional Study. *P R Health Sci J.* (2018) 37:115-23. doi:

2. Oyaro B, Lokken E, Alumera H, Hussein S, Richardson B, Mandaliya K, et al. Prevalence and correlates of periodontitis among Kenyan women planning to conceive. *BMC Oral Health.* (2022) 22:216. doi: 10.1186/s12903-022-02243-w

3. Pihlstrom BL, Michalowicz BS, Johnson NW. Periodontal diseases. *Lancet.* (2005) 366:1809-20. doi: 10.1016/S0140-6736(05)67728-8

4. Schievelbein BS, Casarin RP, da Mota Kruger MS, CF ES, da Silveira TM, Romano AR, et al. Systemic Profile and Periodontal Condition of Hospitalized Women with High-Risk Pregnancy: A Cross-Sectional Study. *Matern Child Health J.* (2023) 27:1264-71. doi: 10.1007/s10995-023-03659-8

5. Kassebaum NJ, Bernabe E, Dahiya M, Bhandari B, Murray CJL, Marcenes W. Global Burden of Severe Periodontitis in 1990-2010: A Systematic Review and Meta-regression. *J Dent Res.* (2014) 93:1045-53. doi: 10.1177/0022034514552491

6. GBD GBoDODC, Bernabe E, Marcenes W, Hernandez CR, Bailey J, Abreu LG, et al. Global, regional, and national levels and trends in burden of oral conditions from 1990 to 2017: a systematic analysis for the Global Burden of Disease 2017 Study. *J Dent Res.* (2020) 99:362-73. doi: 10.1177/0022034520908533

7. GBD GBoDIIPC. Global, regional, and national incidence, prevalence, and years lived with disability for 354 diseases and injuries for 195 countries and territories, 1990-2017: a systematic analysis for the Global Burden of Disease Study 2017. *Lancet.* (2018) 392:1789-858. doi: 10.1016/S0140-6736(18)32279-7

8. Chen MX, Zhong YJ, Dong QQ, Wong HM, Wen YF. Global, regional, and national burden of severe periodontitis, 1990-2019: An analysis of the Global Burden of Disease Study 2019. *J Clin Periodontol.* (2021) 48:1165-88. doi: 10.1111/jcpe.13506

9. Zhang X, Wang X, Wu J, Wang M, Hu B, Qu H, et al. The global burden of periodontal diseases in 204 countries and territories from 1990 to 2019. *Oral Dis.* (2022) n/a. doi: 10.1111/odi.14436

10. Wu L, Zhang SQ, Zhao L, Ren ZH, Hu CY. Global, regional, and national burden of periodontitis from 1990 to 2019: Results from the Global Burden of Disease study 2019. *J Periodontol.* (2022) 93:1445-54. doi: 10.1002/JPER.21-0469

11. Eke PI, Page RC, Wei L, Thornton-Evans G, Genco RJ. Update of the case definitions for population-based surveillance of periodontitis. *J Periodontol.* (2012) 83:1449-54. doi: 10.1902/jop.2012.110664

12. Eke PI, Thornton-Evans GO, Wei L, Borgnakke WS, Dye BA, Genco RJ. Periodontitis in US Adults: National Health and Nutrition Examination Survey 2009-2014. *J Am Dent Assoc.* (2018) 149:576-88 & 88.e1-88.e6. doi: 10.1016/j.adaj.2018.04.023

13. Zhang Y, Leveille SG, Shi L, Camhi SM. Disparities in Preventive Oral Health Care and Periodontal Health Among Adults With Diabetes. *Prev Chronic Dis.* (2021) 18:E47. doi: 10.5888/pcd18.200594

14. Eke PI, Wei L, Borgnakke WS, Thornton-Evans G, Zhang X, Lu H, et al. Periodontitis prevalence in adults >/= 65 years of age, in the USA. *Periodontol 2000.* (2016) 72:76-95. doi: 10.1111/prd.12145

15. Righolt AJ, Jevdjevic M, Marcenes W, Listl S. Global-, Regional-, and Country-Level Economic Impacts of Dental Diseases in 2015. *J Dent Res.* (2018) 97:501-07. doi: 10.1177/0022034517750572

16. Botelho J, Machado V, Leira Y, Proença L, Chambrone L, Mendes JJ. Economic burden of periodontitis in the United States and Europe - an updated estimation. *J Periodontol.* (2022) 93:373-79. doi: 10.1002/JPER.21-0111

17. Sato M, Iwasaki M, Yoshihara A, Miyazaki H. Association between periodontitis and medical expenditure in older adults: A 33-month follow-up study. *Geriatr Gerontol Int.* (2016) 16:856-64. doi: 10.1111/ggi.12569

18. Lamster IB, Malloy KP, DiMura PM, Cheng B, Wagner VL, Matson J, et al. Dental Services and Health Outcomes in the New York State Medicaid Program. *J Dent Res.* (2021) 220345211007448. doi: 10.1177/00220345211007448

19. Mendes JJ, Botelho J, Machado V, Klinge B, Alves de Matos AP, Alcoforado G. Oral health and public health: the effects and costs of Periodontitis. EuroHealthNet Magazine,, 2021.11.25.

20. NADP. NADP Analysis Shows Adults with Medicaid Preventive Dental Benefits Have Lower Medical Costs for Chronic Conditions. 2017.

21. Ramseier CA, Manamel R, Budmiger R, Cionca N, Sahrmann P, Schmidlin PR, et al. Cost savings in the Swiss healthcare system resulting from professional periodontal care. *Swiss dental journal.* (2022) 132:764-79. doi:

22. Sun H, Saeedi P, Karuranga S, Pinkepank M, Ogurtsova K, Duncan BB, et al. IDF Diabetes Atlas: Global, regional and country-level diabetes prevalence estimates for 2021 and projections for 2045. *Diabetes Res Clin Pract.* (2022) 183:109119. doi: 10.1016/j.diabres.2021.109119

23. Magliano DJ, Boyko EJ. IDF Diabetes Atlas; 10th ed. Available: https://www.diabetesatlas.org/en/. IDF DIABETES ATLAS. Brussels: International Diabetes Federation

© International Diabetes Federation, 2021., 2021.

24. Saeedi P, Petersohn I, Salpea P, Malanda B, Karuranga S, Unwin N, et al. Global and regional diabetes prevalence estimates for 2019 and projections for 2030 and 2045: Results from the International Diabetes Federation Diabetes Atlas, 9(th) edition. *Diabetes Res Clin Pract.* (2019) 157:107843. doi: 10.1016/j.diabres.2019.107843

25. Centers for Disease Control and Prevention (CDC). National Diabetes Statistics Report, 2020. Atlanta, GA: Centers for Disease Control and Prevention. National Diabetes Statistics Report website. https://www.cdc.gov/diabetes/data/statistics-report/index.html. Accessed [date]. 2020:32. https://www.cdc.gov/diabetes/pdfs/data/statistics/national-diabetes-statistics-report.pdf.

26. Bommer C, Heesemann E, Sagalova V, Manne-Goehler J, Atun R, Barnighausen T, et al. The global economic burden of diabetes in adults aged 20-79 years: a cost-of-illness study. *The lancet Diabetes & endocrinology.* (2017). doi: 10.1016/S2213-8587(17)30097-9

27. Squires E, Duber H, Campbell M, Cao J, Chapin A, Horst C, et al. Health Care Spending on Diabetes in the U.S., 1996-2013. *Diabetes Care.* (2018) 41:1423-31. doi: 10.2337/dc17-1376

28. American Diabetes Association. Economic Costs of Diabetes in the U.S. in 2017. *Diabetes Care.* (2018) 41:917-28. doi: 10.2337/dci18-0007

29. Dall TM, Yang W, Gillespie K, Mocarski M, Byrne E, Cintina I, et al. The Economic Burden of Elevated Blood Glucose Levels in 2017: Diagnosed and Undiagnosed Diabetes, Gestational Diabetes Mellitus, and Prediabetes. *Diabetes Care.* (2019) 42:1661-68. doi: 10.2337/dc18-1226

30. American Diabetes Association (AD(M)A). Cost of diabetes. 2022.

31. Shrestha SS, Honeycutt AA, Yang W, Zhang P, Khavjou OA, Poehler DC, et al. Economic Costs Attributable to Diabetes in Each U.S. State. *Diabetes Care.* (2018) 41:2526-34. doi: 10.2337/dc18-1179

32. Weber MB, Narayan KMV. Health Insurance for Diabetes Prevention Confers Health Benefits and Breaks Even on Cost Within 2 Years. *Diabetes Care.* (2019) 42:1612-14. doi: 10.2337/dci19-0022

33. O'Connell JM, Manson SM. Understanding the Economic Costs of Diabetes and Prediabetes and What We May Learn About Reducing the Health and Economic Burden of These Conditions. *Diabetes Care.* (2019) 42:1609-11. doi: 10.2337/dci19-0017

34. Darling-Fisher CS, Borgnakke WS, Haber J. Oral health and diabetes; Gain the confidence to discuss this important topic with your patients. *Am Nurse Today.* (2017) 12:22-25. doi: n/a

35. Darling-Fisher CS, Kanjirath PP, Peters MC, Borgnakke WS. Oral health: an untapped resource in managing glycemic control in diabetes and promoting overall health. *The Journal for Nurse Practitioners.* (2015) 11:889–96. doi: 10.1016/j.nurpra.2015.08.001, Available:

https://www.npjournal.org/article/S1555-4155(15)00735-7/pdf

36. Buset SL, Walter C, Friedmann A, Weiger R, Borgnakke WS, Zitzmann NU. Are periodontal diseases really silent? A systematic review of their effect on quality of life. *J Clin Periodontol.* (2016) 43:333-44. doi: 10.1111/jcpe.12517

37. Sanz M, Ceriello A, Buysschaert M, Chapple I, Demmer RT, Graziani F, et al. Scientific evidence on the links between periodontal diseases and diabetes: Consensus report and guidelines of the joint workshop on periodontal diseases and diabetes by the International diabetes Federation and the European Federation of Periodontology. *Diabetes Res Clin Pract.* (2018b) 137:231-41. doi: 10.1016/j.diabres.2017.12.001

38. Sanz M, Ceriello A, Buysschaert M, Chapple I, Demmer RT, Graziani F, et al. Scientific evidence on the links between periodontal diseases and diabetes: Consensus report and guidelines of the joint workshop on periodontal diseases and diabetes by the International Diabetes Federation and the European Federation of Periodontology. *J Clin Periodontol.* (2018a) 45:138-49. doi: 10.1111/jcpe.12808

39. Iwasaki M, Usui M, Ariyoshi W, Nakashima K, Nagai-Yoshioka Y, Inoue M, et al. Validation of a self-report questionnaire for periodontitis in a Japanese population[Note:AuthorCOrrection: PMID:34462529]. *Scientific reports.* (2021) 11:15078. doi: 10.1038/s41598-021-93965-4

40. Montero E, La Rosa M, Montanya E, Calle-Pascual AL, Genco RJ, Sanz M, et al. Validation of self-reported measures of periodontitis in a Spanish Population. *J Periodontal Res.* (2020) 55:400-09. doi: 10.1111/jre.12724

41. Saka-Herran C, Jane-Salas E, Gonzalez-Navarro B, Estrugo-Devesa A, Lopez-Lopez J. Validity of a self-reported questionnaire for periodontitis in Spanish population[NOTE: Cites Page, but uses Eke perio definitions, "mild"]. *J Periodontol.* (2020) n/a. doi: 10.1002/JPER.19-0604

42. Micu IC, Bolboacă SD, Caracostea GV, Gligor D, Ciurea A, Iozon S, et al. Self-reported and clinical periodontal conditions in a group of Eastern European postpartum women. *PLoS One.* (2020) 15:e0237510. doi: 10.1371/journal.pone.0237510

43. Miller K, Eke PI, Schoua-Glusberg A. Cognitive evaluation of self-report questions for surveillance of periodontitis. *J Periodontol.* (2007) 78:1455-62. doi: 10.1902/jop.2007.060384

44. Eke PI, Dye B. Assessment of self-report measures for predicting population prevalence of periodontitis. *J Periodontol.* (2009) 80:1371-9. doi: 10.1902/jop.2009.080607

45. Eke PI, Dye BA, Wei L, Slade GD, Thornton-Evans GO, Beck JD, et al. Self-reported measures for surveillance of periodontitis. *J Dent Res.* (2013) 92:1041-7. doi: 10.1177/0022034513505621

46. Verhulst MJL, Teeuw WJ, Gerdes VEA, Loos BG. Self-reported oral health and quality of life in patients with type 2 diabetes mellitus in primary care: a multi-center cross-sectional study. *Diabetes, metabolic syndrome and obesity : targets and therapy.* (2019) 12:883-99. doi: 10.2147/DMSO.S207087

47. Nijland N, Overtoom F, Gerdes VEA, Verhulst MJL, Su N, Loos BG. External validation of a rapid, non-invasive tool for periodontitis screening in a medical care setting. *Clin Oral Investig.* (2021). doi: 10.1007/s00784-021-03952-2

48. Verhulst MJL, Teeuw WJ, Bizzarro S, Muris J, Su N, Nicu EA, et al. A rapid, non-invasive tool for periodontitis screening in a medical care setting. *BMC Oral Health.* (2019) 19:87. doi: 10.1186/s12903-019-0784-7

49. Verhulst MJL, Teeuw WJ, Gerdes VEA, Loos BG. Implementation of an Oral Care Protocol for Primary Diabetes Care: A Pilot Cluster-Randomized Controlled Trial. *Annals of family medicine.* (2021) 19:197-206. doi: 10.1370/afm.2645

50. Verhulst MJL. Implementation of oral care in primary diabetes care [Cited Refs in Chapter 6: Summary, discussion and conclusion]. Amsterdam: Universiteit van Amsterdam, 2019.

51. George A, Poudel P, Kong A, Villarosa A, Calache H, Arora A, et al. Developing and pilot testing an oral health screening tool for diabetes care providers. *BMC Prim Care.* (2022) 23:202. doi: 10.1186/s12875-022-01798-5

52. Sekundo C, Bölk T, Kalmus O, Listl S. Accuracy of a 7-Item Patient-Reported Stand-Alone Tool for Periodontitis Screening. *Journal of clinical medicine.* (2021) 10:287. doi: 10.3390/jcm10020287

53. Tonetti MS, Deng K, Christiansen A, Bogetti K, Nicora C, Thurnay S, et al. Self-reported bleeding on brushing as a predictor of bleeding on probing: Early observations from the deployment of an internet of things network of intelligent power-driven toothbrushes in a supportive periodontal care population. *J Clin Periodontol.* (2020) 47:1219-26. doi: 10.1111/jcpe.13351
